# In Vitro Analysis of Interactions Between *Staphylococcus aureus* and *Pseudomonas aeruginosa* During Biofilm Formation

**DOI:** 10.3390/antibiotics14050504

**Published:** 2025-05-14

**Authors:** Julia Scaffo, Rayssa Durães Lima, Cameron Dobrotka, Tainara A. N. Ribeiro, Renata F. A. Pereira, Daniela Sachs, Rosana B. R. Ferreira, Fabio Aguiar-Alves

**Affiliations:** 1 Molecular Epidemiology and Biotechnology Laboratory, Fluminense Federal University, Niteroi 24241-000, Brazil; 2 Postgraduate Program in Sciences Applied to Health Products, Fluminense Federal University, Niteroi 24241-000, Brazil; 3 Instituto de Microbiologia Paulo de Goes, Federal University of Rio de Janeiro, Rio de Janeiro 21941-902, Brazil; rayssalima2401@gmail.com (R.D.L.);; 4 Molecular Biosciences Department, The University of Kansas, Lawrence, KS 66045, USA; 5 Department of Pharmaceutical Sciences, Lloyd L. Gregory School of Pharmacy, Palm Beach Atlantic University, West Palm Beach, FL 33401, USA; cameron_dobrotka@pba.edu; 6 Postgraduate Program in Engineering Materials, Institute of Physics and Chemistry, Federal University of Itajuba, Itajuba 37500-903, Brazildanisachs@unifei.edu.br (D.S.); 7 Postgraduate Program in Applied Microbiology and Parasitology, Fluminense Federal University, Niteroi 24210-130, Brazil; 8 Postgraduate Program in Pathology, Fluminense Federal University, Niteroi 24033-900, Brazil

**Keywords:** *Staphylococcus aureus*, *Pseudomonas aeruginosa*, polymicrobial biofilms, antimicrobial resistance, ESKAPE

## Abstract

*Staphylococcus aureus* and *Pseudomonas aeruginosa* are classified as ESKAPE pathogens that present a significant challenge to treatment due to their increased resistance to a considerable number of antimicrobial agents. Background/Objective**:** Biofilms exacerbate treatment challenges by providing enhanced antimicrobial and environmental protection. Mixed-species biofilms further complicate treatment options through numerous complex interspecies interactions, leading to potentially severe adverse clinical outcomes. Methods: This study assessed the interaction between clinical *S. aureus* and *P. aeruginosa* isolates during biofilm formation using microplate biofilm formation assays, scanning electron microscopy, and confocal microscopy. Results: We identified a competitive relationship between *P. aeruginosa* and *S. aureus*, where both pathogens exhibited a reduction in biofilm formation during mixed-species biofilms compared with monocultures, although *P. aeruginosa* outcompeted *S. aureus*. Furthermore, we found that the cell-free conditioned media (CFCM) of *P. aeruginosa* significantly reduced the *S. aureus* biofilms. Using fractioned CFCM, we identified that the anti-staphylococcal activity of the >10 kDa fraction was almost identical to the non-fractioned CFCM. Our confocal microscopy results suggest that *P. aeruginosa* CFCM depolarize *S. aureus* membranes and reduces the biofilm burden. Conclusions: These findings contribute to our understanding of the mechanisms underlying the interactions between these pathogens, suggesting that there is an antagonistic relationship between *S. aureus* and *P. aeruginosa* in a biofilm setting.

## 1. Introduction

*Staphylococcus aureus* is a Gram-positive, facultative anaerobic bacterium found in the human microbiota, mainly associated with the skin and nasopharynx sites. It is frequently responsible for human infections that range from uncomplicated skin infections to more serious and difficult-to-treat invasive infections [1]. *Pseudomonas aeruginosa* is a motile Gram-negative bacterium considered one of the primary opportunistic human pathogens, affecting mainly immunocompromised patients, especially those with neutropenia, burns, cancer, diabetes mellitus, obstructive pulmonary disease, and cystic fibrosis [2,3]. *S. aureus* and *P. aeruginosa* are classified as ESKAPE (*Enterococcus faecium*, *Staphylococcus aureus*, *Klebsiella pneumoniae*, *Acinetobacter baumannii*, *Pseudomonas aeruginosa*, *Enterobacter* species) pathogens, exhibiting widespread antimicrobial resistance, including multidrug-resistant (MDR) strains, leading to heightened nosocomial and community infections worldwide [4,5]. *S. aureus* and *P. aeruginosa*, in particular, are also frequently co-isolated from certain infection niches, predominantly the skin and the lungs, especially in cases of chronic wounds and cystic fibrosis [6]. Co-infections often lead to the formation of mixed biofilms, which may enhance resistance not only to antimicrobial agents but also to the host’s immune defenses [7]. Biofilm-forming organisms are characterized by their ability to adapt to numerous different environments, often attributed to their metabolic adaptability [8]. Much of this metabolic and environmental adaptation comes from a unique bacterial quorum sensing (QS) systems [9]. Bacterial QS systems, harbored by *S. aureus* and *P. aeruginosa*, respond to environmental stimuli, resulting in altered gene expression and regulating biofilm formation, metabolic pathways, virulence, and motility, among other significant pathways [6,10,11,12]. It is well-documented that polymicrobial biofilm infections are worse than single-species biofilms due to increased persistence and higher antimicrobial resistance through QS systems [13]. As a result, the infection phase can progress into a persistent chronic condition that is difficult to treat. Furthermore, polymicrobial biofilms contribute to the increased recalcitrance to conventional treatments [14].

During polymicrobial infections, *P. aeruginosa* and *S. aureus* interact with one another, often to the detriment of one species, while worsening clinical outcomes [15]. The disharmonic relationship between the two bacterial strains stimulates the production of numerous secreted factors including antibacterial proteins, small molecules, and proteases [10,16,17,18]. Excreted small molecules, such as pyoverdine, have a high affinity for iron in mixed biofilm conditions, sequestering iron and damaging *S. aureus* cell viability [19,20,21]. Additionally, *P. aeruginosa* produces 2-n-heptyl-4-hydroxyquinoline-N-oxide (HQNO), a quorum-sensing agent that has been shown to interfere with the cytochrome *bc1* complex, inhibiting the electron transport chain and proving toxic to several microorganisms including *S. aureus* [22,23]. Furthermore, *P. aeruginosa* secretes an abundance of proteins, including numerous pyocins, functioning as DNases, pore-formers, and RNases [18]. Pyocins have been shown to possess antibiofilm activity against other strains of *P. aeruginosa*, although little is known about the antibiofilm properties against other bacteria [24]. The abundance of excreted *P. aeruginosa* factors results in a hostile environment for other bacteria including *S. aureus*.

In this study, we aimed to evaluate the dynamic interactions between *S. aureus* and *P. aeruginosa* during the process of biofilm formation in vitro. Namely, we wanted to understand how interactions between different strains of *S. aureus* and *P. aeruginosa* influenced the biofilm structure, which could have implications for managing biofilm-associated infections involving these two microorganisms.

## 2. Results

### 2.1. Polymicrobial Biofilm Strength

**Figure 1:**
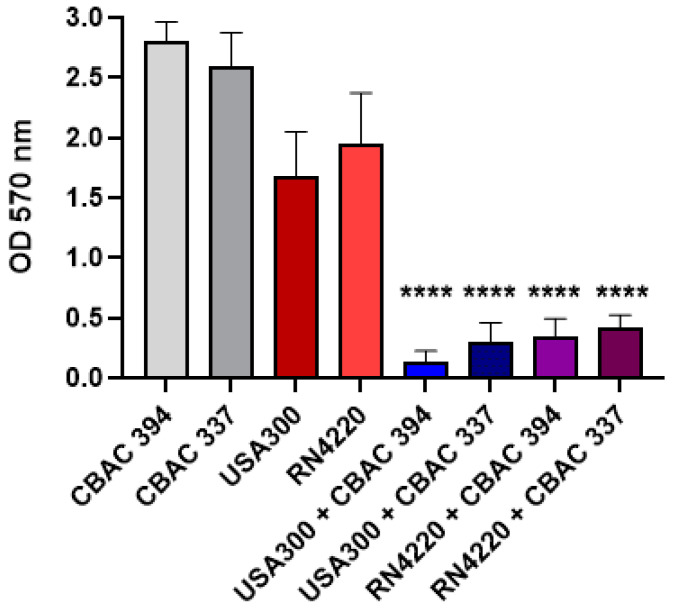
Polymicrobial biofilm formation of *S. aureus* and *P. aeruginosa*. PA 394 and PA 337: *P. aeruginosa* clinical strains; RN4220: Methicillin-susceptible *S. aureus*; USA300: Methicillin-resistant *S. aureus* (n = 3). Polymicrobial biofilms tested included: USA300 + CBAC 394 (i.e., blue bar), USA300 + CBAC 337 (i.e., dark blue bar), RN4220 + CBAC 394 (i.e., purple bar), and RN4220 + CBAC 337 (i.e., plum bar). Statistical analysis was conducted using an ordinary one-way ANOVA with Tukey’s multiple comparisons post hoc test with 95% (*p* < 0.05) significance. The asterisks represent the significant difference between each monoculture and the polymicrobial cultures, respectively. ****: *p* ≤ 0.0001.

To evaluate the impact of polymicrobial interactions of *S. aureus* and *P. aeruginosa* in biofilms, we began by culturing both species together and quantifying the total biofilm mass produced in comparison to monocultures by crystal violet staining. The results showed that for both strains of each species, when *S. aureus* was inoculated with *P. aeruginosa*, there was a significant reduction in the total biofilm mass compared with the monoculture biofilms in both methicillin-resistant *S. aureus* (MRSA; USA 300) and methicillin-susceptible *S. aureus* (MSSA; RN4220), while there was also a significant reduction compared with *P. aeruginosa* CBAC 394 and 337 as the controls (Figure 1).

**Figure 2:**
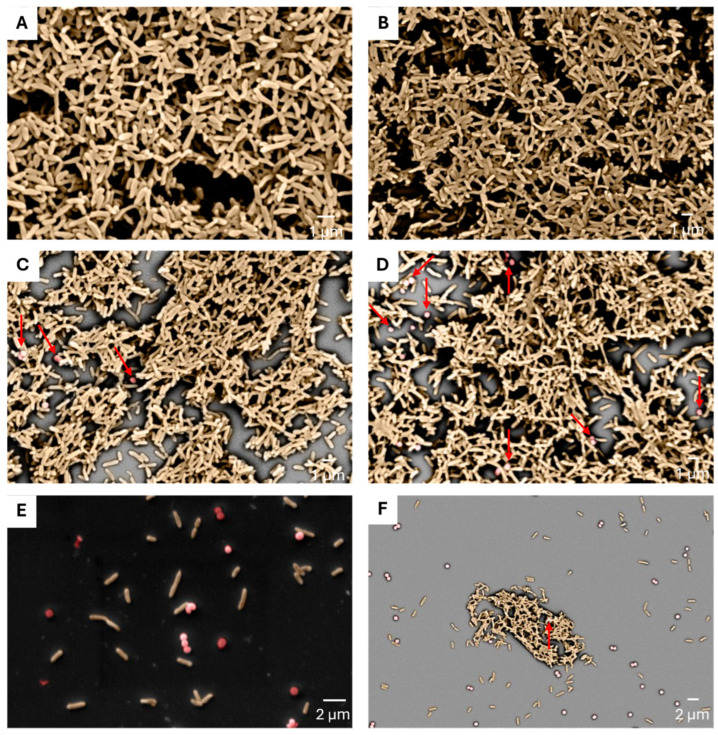
Scanning electron microscopy shows the competitive dynamics between *S. aureus* and *P. aeruginosa* in polymicrobial biofilms. *P. aeruginosa* was digitally colored in gold, while *S. aureus* was colored red. Panels (**A**,**B**) display monocultures of the *P. aeruginosa* CBAC 337 and CBAC 394 strains, respectively. Panels (**C**,**D**) depict polymicrobial biofilms of *P. aeruginosa* CBAC 337 and 394 with *S. aureus* RN4220, while panels (**E**,**F**) show *P. aeruginosa* CBAC 337 and CBAC 394 with *S. aureus* USA300, respectively. Red arrows indicate the location of *S. aureus* cells for clarification and identification purposes.

As we observed a reduction in biofilm mass upon polymicrobial growth, we utilized scanning electron microscopy (SEM) to further dissect the interactions between *S. aureus* and *P. aeruginosa*. We observed different behaviors of *P. aeruginosa* strains in co-culture with different *S. aureus* strains. As expected, both *P. aeruginosa* strains showed robust biofilm growth in the monocultures (Figure 2A,B). However, upon cultivation with *S. aureus* strains, changes in the biofilm structures were observed. Specifically, *P. aeruginosa* CBAC 337 biofilms with *S. aureus* RN4220 showed a predominance of *P. aeruginosa*, with minimal Gram-positive cocci observed, while with *P. aeruginosa* CBAC 394, RN4220 cells were present, although significantly reduced (Figure 2C,D).

Furthermore, the biofilms of both CBAC 337 and 394 were slightly more dispersed and less robust compared with the monocultures (Figure 2C,D). Most notably, we observed a substantial reduction in the number of layers captured. The monoculture controls had substantial growth and numerous layers of *P. aeruginosa* stacking onto one another (Figure 2A,B). In polymicrobial biofilms with *S. aureus* RN4220, there were only a few layers of *P. aeruginosa* captured with limited stacking and a sizable amount of background present, further corroborating our initial biofilm biomass experiments, as presented in Figure 1 (Figure 2C,D).

On the other hand, polymicrobial biofilms of *P. aeruginosa* and *S. aureus* USA300 were dramatically different compared with *P. aeruginosa* monomicrobial biofilms, with a significant reduction in the visible biofilm formed (Figure 2E,F). No biofilm structure was observed when *P. aeruginosa* CBAC 337 and *S. aureus* USA300 were co-cultured, while a minimal biofilm structure formed almost exclusively of CBAC 394 was observed (Figure 2E,F).

Quantification of colony-forming units (CFUs) was performed by using selective media plates. The mixed media control was grown on non-selective plates to quantify the total bacterial abundance. Our results revealed that in co-culture with CBAC 394, USA300 growth was below the limit of detection, while USA300 was significantly reduced (10^7^ CFU/mL) in co-culture with CBAC 337 compared with the co-culture control (Figure S1A,B). Interestingly, CBAC 394 and 337 had no significant reduction in growth when in co-culture with USA300 (Figure S1A,B). Although a slight reduction in growth was observed, this can likely be attributed to growth taking place on selective media plates. Unsurprisingly, in co-culture with both CBAC 394 and CBAC 337, RN4220 was below the limit of detection, while only a slight reduction in CBAC levels was observed (Figure S1C,D).

### 2.2. Inhibition of S. aureus Biofilm Formation and Planktonic Growth with CFCM

**Figure 3:**
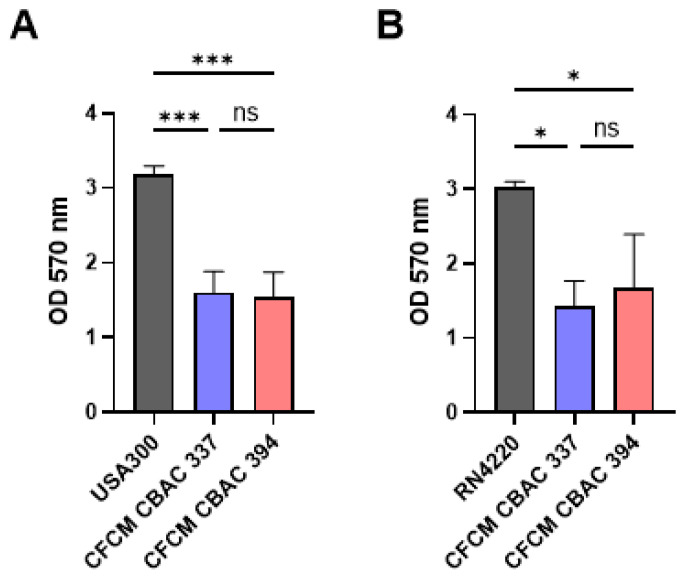
Inhibition of *S. aureus* biofilms in the presence of cell-free conditioned media (CFCM) of *P. aeruginosa*. Biofilms of *S. aureus* USA 300 treated with CFCM of *P. aeruginosa* CBAC 337 and CBAC 394 (**A**) and biofilms of *S. aureus* RN4220 cultivated in the presence of the CFCM of *P. aeruginosa* CBAC 337 and CBAC 394 (**B**) (n = 3). Statistical analysis was conducted using an ordinary one-way ANOVA comparing each sample with one another with Dunnett’s post hoc test with 95% (*p* < 0.05) of significance where *: *p* ≤ 0.05; ***: *p* ≤ 0.001; ns: not significant.

To further explore the lack of *S. aureus* in polymicrobial biofilms with *P. aeruginosa*, we tested the effect of the *P. aeruginosa* cell-free conditioned media (CFCM) on *S. aureus* biofilms. CFCM were extracted from the supernatant of the overnight growth of each *P. aeruginosa* strain (CBAC 337 and 394) and concentrated to test for activity against each *S. aureus* strain. To do so, we performed standard crystal violet staining of the residual bacterial biofilm mass. We found that the biofilm mass of methicillin-resistant *S. aureus* USA300 and methicillin-susceptible *S. aureus* RN4220 was significantly decreased when treated with CFCM from CBAC 337 and 394 compared with the control (Figure 3A,B).

**Figure 4:**
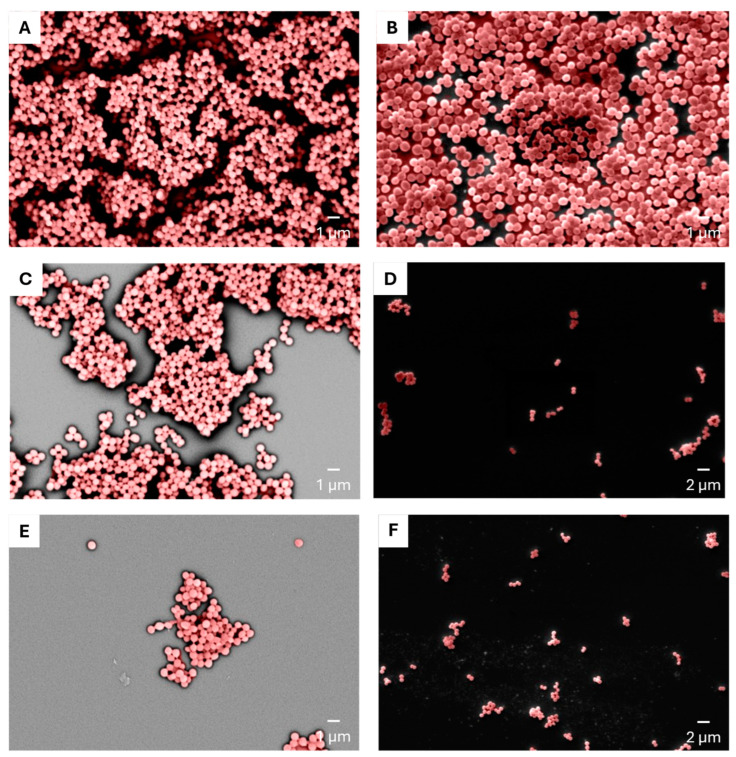
Scanning electron microscopy of the *S. aureus* biofilms treated with cell-free conditioned media (CFCM) of *P. aeruginosa*. Panels (**A**,**B**) represent the controls for *S. aureus* RN4220 and USA300, respectively. Panels (**C**,**D**) show *S. aureus* RN4220 and USA300 grown with CFCM from *P. aeruginosa* CBAC 337, while panels (**E**,**F**) depict the same strains treated with CFCM from *P. aeruginosa* CBAC 394.

Seeing a reduction in the biofilm mass, we hypothesized that the biofilm structure was also likely to be altered. The biofilm structure was evaluated by SEM after treatment with the supernatants from CBAC 337 and CBAC 394 to further investigate how CFCM altered the biofilm structure and density. We found that CBAC 337 altered the RN4220 biofilm structure while limiting the overall density compared with the control (Figure 4A,C). Interestingly, CBAC 337 strongly inhibited any biofilm formation in the methicillin-resistant USA300, with only a sparse bacterial population remaining (Figure 4D).

While CBAC 337 produced substantial anti-biofilm activity, CBAC 394 was more potent than CBAC 337. When treated with CBAC 394 CFCM, RN4220 was unable to form a biofilm (Figure 4E). Similar results were obtained when *S. aureus* USA300 was grown in the presence of CBAC 337 CFCM; limited to no biofilm growth was observed, with some damage to the cellular structure (Figure 4F).

We also detected differences in the number of CFU/mL recovered from the *S. aureus* biofilms after treatment with *P. aeruginosa* CFCM, in agreement with our observations with SEM imaging (Figure 4A–F). Overall, the number of CFU/mL recovered from USA300 and RN4220 biofilms was significantly reduced when treated with CFCM from either CBAC 337 or CBAC 394 (Figure S2A,B). The reduction in CFU/mL was comparable between USA300 treated with either CFCM (Figure S2A). The CFCM derived from CBAC 394 was more potent than CBAC 337 against RN4220, in agreement with our previous results with these CFCM (Figure S2B). Overall, the CFCM from both *P. aeruginosa* strains were capable of significantly reducing the colony-forming units of the *S. aureus* MRSA and MSSA strains.

**Figure 5:**
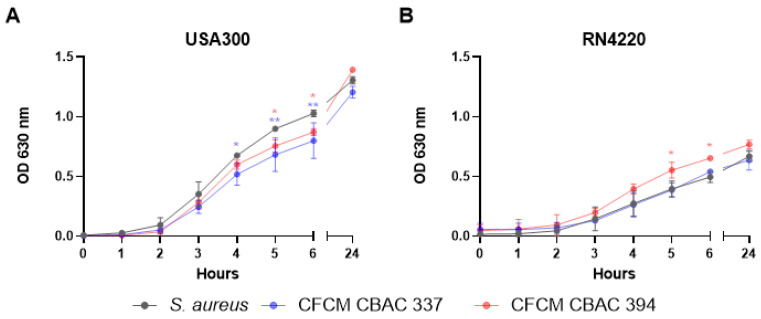
Planktonic growth of *S. aureus* in the presence or absence of *P. aeruginosa* cell-free conditioned media (CFCM). Growth of *S. aureus* USA 300 without treatment and treated with the *P. aeruginosa* CFCM of CBAC 337 and CBAC 394 (**A**). Growth of *S. aureus* RN4220 without treatment and treated with *P. aeruginosa* CFCM of CBAC 337 and CBAC 394 (**B**). CFCM 337: *P. aeruginosa* CBAC 337 cell-free conditioned media; CFCM 394: *P. aeruginosa* CBAC 394 cell-free conditioned media. All results were confirmed through CFU/mL enumeration (n = 3). Statistical analysis was conducted using two-way ANOVA with the Dunnett post hoc test with 95% (*p* < 0.05) significance. *: *p* ≤ 0.05; **: *p* ≤ 0.01.

Given the impact of the *P. aeruginosa* CFCM on *S. aureus* biofilm formation, we evaluated their effect on *S. aureus* planktonic growth using a standard growth curve assay. Here, we found that CFCM from both *P. aeruginosa* strains slightly but significantly reduced the growth of *S. aureus* USA300 (Figure 5A). For the CBAC 337 CFCM, we were able to see a significant reduction in the growth of USA300 between the fourth and sixth hours of growth (Figure 5A). For the CBAC 394 CFCM, a significant reduction in the growth rate of USA300 was only seen after five hours of growth (Figure 5A). After 24 h, there was no difference in the growth rates with either CFCM. When the same experiments were conducted on MSSA strain RN4220, no growth inhibition was observed for either *P. aeruginosa* CFCM (Figure 5B). The CBAC 394 CFCM appeared to increase the growth of RN4220 slightly, which was evident after three hours of growth (Figure 5B).

### 2.3. Fractionation of CFCM Activity by Molecular Weight and Impact on Growth

**Figure 6:**
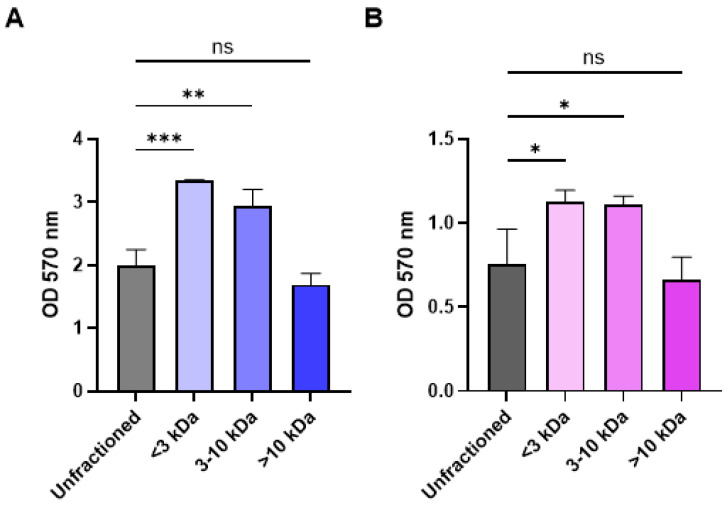
Treatment of *S. aureus* biofilms with the CFCM of *P. aeruginosa* CBAC 394 fractionated into different molecular weights (>10 kDa, between 3-10 kDa and < 3 kDa) against *S. aureus* (**A**) USA300 and (**B**) RN4220. Unfractionated CFCM of CBAC 394 were used as the control (n = 3). Statistical analysis was conducted using an ordinary one-way ANOVA with the Dunnett post hoc test with 95% (*p* < 0.05) significance. *: *p* ≤ 0.05; **: *p* ≤ 0.01; ***: *p* ≤ 0.001; ns, not significant.

Since the CBAC 394 CFCM was more effective in reducing methicillin-resistant *S. aureus* USA300 and methicillin-susceptible *S. aureus* RN4220 biofilms, we wanted to identify a relative molecular weight for the molecules responsible for CFCM activity. For this, CFCM from CBAC 394 was fractionated using a molecular weight filter, separating the CFCM into three groups: <3 kDa, 3–10 kDa, and >10 kDa. We found that when compared with the unfractionated control, only the fraction of CFCM > 10 kDa provided similar results against both *S. aureus* USA300 (methicillin-resistant) and RN4220 (methicillin-susceptible) (Figure 6). On the contrary, neither CFCM < 10 kDA provided any appreciable activity, as there was no reduction in growth after fractionation (Figure 6).

### 2.4. Growth in Polymicrobial Biofilm Increases Expression of Virulence Factors and Excreted Proteins

**Figure 7:**
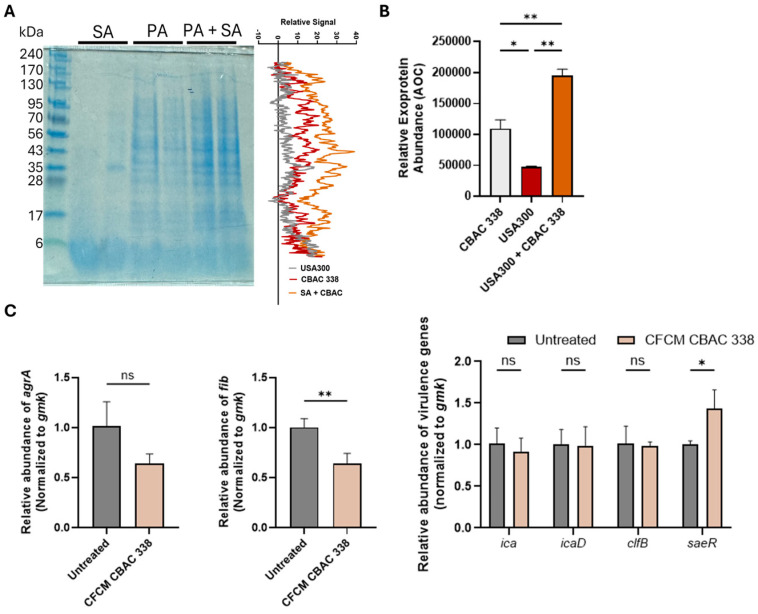
Effects of the polymicrobial biofilm on the (**A**) exoprotein production in the monomicrobial biofilm (SA: USA300; PA: CBAC 338; PA + SA: CBAC 338 + USA300) and polymicrobial biofilm with (**B**) protein abundance quantified by the area under the curve (AOC) analysis (n = 2). The effects of cell-free conditioned media (CFCM) on *S. aureus* virulence gene expression after 24 h of treatment (n = 3) (**C**). Statistical analysis was conducted using an ordinary one-way ANOVA with the Dunnett post hoc test with 95% (*p* < 0.05) significance, Welch’s *t*-test (*p* < 0.05) of significance, and two-way ANOVA with Šídák’s multiple comparisons test. *: *p* ≤ 0.05; **: *p* ≤ 0.01; ns, not significant.

After identifying that active components produced by CBAC 394 were larger than 10 kDA, we wanted to analyze the production of extracellular proteins when *P. aeruginosa* and *S. aureus* were in a polymicrobial biofilm compared with monomicrobial biofilm. To do so, methicillin-resistant *S. aureus* USA300 and *P. aeruginosa* CBAC 338 were grown individually and in a polymicrobial biofilm in 6-well plates for 24 h. After 24 h, the supernatant was collected and normalized by OD_600_. The supernatant was assessed by SDS-PAGE electrophoresis followed by Coomassie staining. Unsurprisingly, the CFCM activity was only observed at >10 kDa, as all prominent secreted protein bands seen in both CBAC 338 and polymicrobial biofilm appeared to be larger than 10 kDa (Figure 7A). Interestingly, it appears that in the polymicrobial biofilms, the abundance of CBAC 338 excreted proteins was higher compared with CBAC 338 alone (Figure 7B). Importantly, the protein bands observed in the *S. aureus* monomicrobial biofilms were not seen in the polymicrobial biofilms (Figure 7A).

We further wanted to examine the role of *P. aeruginosa* CFCM on the expression of key virulence factors involved in methicillin-resistant *S. aureus* USA300 biofilm formation. We postulated that the increase in exoprotein abundance would likely affect *S. aureus* virulence factor gene expression. We chose to examine the following biofilm-associated virulence factors as they are integral to *S. aureus* biofilm formation and virulence within the biofilm: *agrA*, *fib*, *ica*, *icaD*, *clfB*, and *saeR* [25,26,27]. We observed that upon treatment with CFCM, a few key virulence factors were identified to be differentially expressed including factors of two two-component systems and key attachment molecules. Specifically, we observed the repression of *agr*A (two-component system) and *fib* (attachment) gene expression in the presence of *P. aeruginosa* CBAC 338 CFCM (Figure 7C). We also observed an increase in *sae*R, a member of the *S. aureus* exoprotein regulatory network [28]. Unsurprisingly, there was no change in the expression of the *ica* operon as the *ica* operon does not influence biofilm formation, and thus we would not expect any change upon treatment with CFCM [29].

### 2.5. P. aeruginosa Excreted Proteins Eliminate S. aureus Biofilms

#### 2.5.1. *P. aeruginosa* Kills *S. aureus* During Polymicrobial Growth Through Membrane Depolarization

As we identified, *P. aeruginosa* strains are effective in killing *S. aureus* bacteria in a polymicrobial setting, so we also investigated a possible mechanism of action for *S. aureus* cell death. Antimicrobial peptides (AMPs) have been previously developed as therapeutics, while the human body inherently produces AMPs [30]. AMPs are usually considered to disrupt bacterial membranes, causing depolarization and eventual lysis due to osmotic pressure. To investigate whether this was the mechanism by which *P. aeruginosa* eliminates *S. aureus*, we used the membrane potential-sensitive dye DiBAC4 and monitored dual-species biofilms via confocal microscopy to assess changes in membrane depolarization.

**Figure 8:**
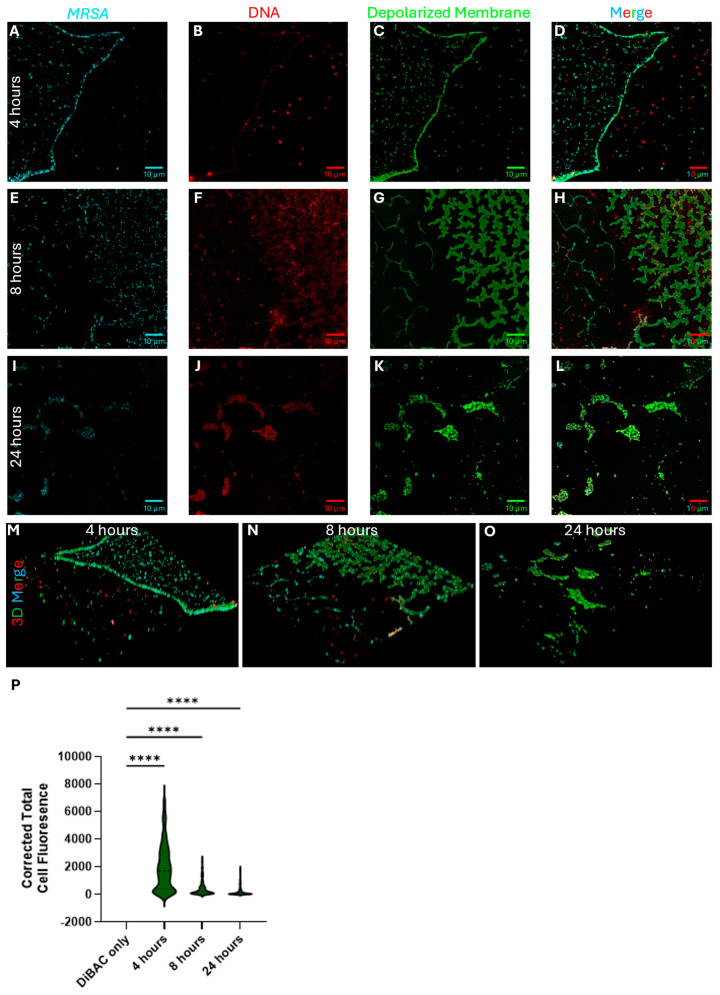
Co-culture of *S. aureus* MRSA and *P. aeruginosa* biofilms at 4 h (**A**–**D**), 8 h (**E**–**H**), and 24 h (**I**–**L**). Wheat Germ Agglutinin Alexa Fluor 647 was used to identify *S. aureus* cells (**A**,**E**,**I**) while SYTO 84 was used to label both intracellular and extracellular DNA (**B**,**F**,**J**) including eDNA in the matrix of the biofilm. Panels (**C**,**G**,**K**) show membrane depolarization using DiBAC4, a voltage-sensitive fluorophore. Panels (**D**,**H**,**L**) are merged images of all fluorophores used, showing overlap between *S. aureus* cells and cells that were depolarized, which was further seen throughout the entire biofilm (**M**–**O**). The corrected total cell fluorescence was calculated to further show the depolarization present (**P**). Statistical analysis was performed by one-way ANOVA with Dunnett’s multiple comparison test with 95% (*p* < 0.05) significance. ****: *p* ≤ 0.0001.

We grew methicillin-resistant *S. aureus* USA300 and *P. aeruginosa* CBAC 338 on glass coverslips in a 1:1 ratio in BHI broth with the addition of 1% glucose for 4, 8, and 24 h to assess the changes in membrane depolarization and composition over time. We used three fluorescent dyes, DiBAC4, SYTO84, and Wheat Germ Agglutinin Alexa Fluor 647 staining, for membrane depolarization, intra- and extracellular DNA, and *S. aureus*, respectively. We visualized each of these samples right after staining was completed by confocal microscopy under oil immersion (Figure 8). At the four-hour mark of the initial biofilm formation (Figure 8A–D), we observed a mixture of both *S. aureus* and *P. aeruginosa*, although the sample contained predominantly *S. aureus*. Many of the *S. aureus* cells were already depolarized at this stage. Interestingly, most SYTO84 signals (DNA) were localized within the cells themselves, with limited diffusion to the extracellular area. Many of the present *P. aeruginosa* cells were also depolarized, as shown by the characteristic green color. Notably, *P. aeruginosa* appeared to form a separate biofilm at the air–liquid interface rather than on the coverslip surface, which was predominantly colonized by *S. aureus*. It also appeared that *S. aureus* was beginning to form some semblance of structure, as a line of *S. aureus* can be observed, forming what is likely an outline of a structured biofilm that would later be formed.

After 8 h, we observed a significant change in the cell dynamics including those that were depolarized (Figure 8E–H). There were limited amounts of *P. aeruginosa* present on the glass coverslip. The *P. aeruginosa* biofilm again appeared at the air–liquid interface and was removed during washing, rather than colonizing the coverslip, which was dominated by *S. aureus*. Even though the coverslip was dominated by *S. aureus*, cells remaining attached were depolarized and soon to be lysed. There were observable structures that could be made out with *S. aureus* cells present in the structures, but all remaining structures were depolarized (Figure 8G). Interestingly, there was a significant amount of extracellular DNA (eDNA) present after 8 h, something not seen in early development and late-stage incubation. After 24 h of co-incubation, there were few to no *S. aureus* cells remaining, as imaging was conducted using one of a few areas of cell density left. All *S. aureus* cells remaining attached to the coverslip appeared to be depolarized with a reduced amount of eDNA present. *S. aureus* cells that may not have been depolarized were associated with a large density of eDNA. Furthermore, the amount of DiBAC4 fluorescence was calculated for each cell by identifying *S. aureus* cells using the Alexa Fluor 647 signal, followed by measuring the pixel density in the cells in the DiBAC4 channel. Unsurprisingly, we observed a significant increase in the signal intensity between the control and dual-biofilm samples (Figure 8P).

#### 2.5.2. *P. aeruginosa* CFCM Depolarize *S. aureus* Membranes, Leading to Cell Death

**Figure 9:**
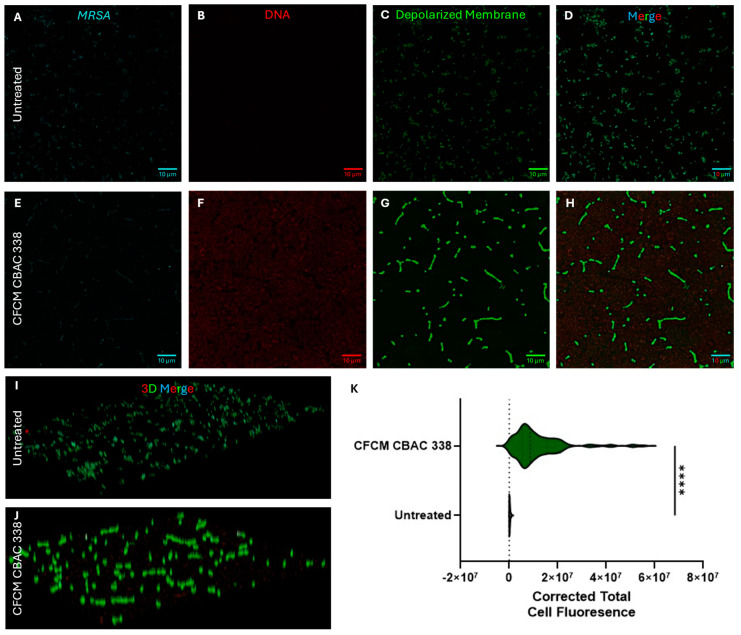
*S. aureus* MRSA biofilms grown without *P. aeruginosa* cell-free conditioned media (CFCM) (**A**–**D**) or with CFCM (**E**,**H**) for 24 h. Wheat Germ Agglutinin Alexa Fluor 647 was used to identify *S. aureus* cells (**A**,**E**), while SYTO 84 was used to label both intracellular and extracellular DNA (**B**,**F**) including eDNA in the matrix of the biofilm. Depolarized membranes were stained using DiBAC4 voltage-sensitive dye (**C**,**G**). All channels were overlaid, showing DiBAC4 staining of the *S. aureus* cells (**D**,**H**), while volume rendering showed that all layers of the *S. aureus* biofilms were depolarized upon treatment of CFCM (**I**–**J**). Membrane depolarization was quantified using corrected total cell fluorescence (**K**). Statistical analysis was performed by one-way ANOVA with Dunnett’s multiple comparison test with 95% (*p* < 0.05) significance. ****: *p* ≤ 0.0001.

After determining that *P. aeruginosa* depolarizes *S. aureus* membranes in co-culture, we wanted to determine whether this held using CFCM. To do so, *S. aureus* biofilms were co-treated with CFCM and incubated for 24 h on glass coverslips. Indeed, the *P. aeruginosa* CFCM were capable of depolarizing *S. aureus* membranes after 24 h of treatment (Figure 9A–K). After 24 h of treatment, there was also an observable increase in eDNA present, both present inside defined cell structures as well as externally, with a marked increase in eDNA corresponding to depolarization (Figure 9B–F). The membranes of the CFCM-treated *S. aureus* were significantly more depolarized than those in the untreated control group (Figure 9C,D,G,H,K).

**Figure 10:**
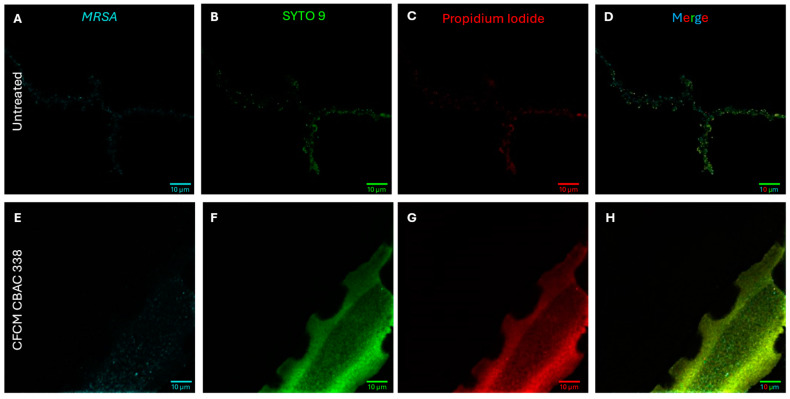
*S. aureus* MRSA biofilms grown without *P. aeruginosa* cell-free conditioned media (CFCM) (**A**–**D**) or with CFCM (**E**–**H**) for 24 h to assess the viability of bacterial cells remaining. Wheat Germ Agglutinin Alexa Fluor 647 was used to identify the *S. aureus* cells (**A**,**E**). Live (**B**,**F**) and dead (**C**,**G**) were visualized using SYTO 9 and propidium iodide, respectively, and captured simultaneously. Merged images were constructed by overlaying all channels to visualize the extent to which *S. aureus* cells were killed by CFCM (**D**,**H**).

Not only are *P. aeruginosa* secreted molecules potent membrane depolarizers, but they are also rather efficient in causing *S. aureus* cell death. Using SYTO 9 and propidium iodide (PI) dyes to assess the amount of cell death caused by the treatment of *P. aeruginosa* CFCM (Figure 10A–H). Much of the signal observed in the PI (dead) channel could be attributed to eDNA making up the biofilm matrix. After 24 h, a majority of cells present were still alive (Figure 10B–D), while minimal cells after treatment with *P. aeruginosa* CFCM were viable (Figure 10F–H) [31]. Cell death, causing the release of eDNA, was also present here, as there was a strong PI signal outside of the cell membrane boundary in the CFCM samples (Figure 10G).

## 3. Discussion

*S. aureus* and *P. aeruginosa* are prominent pathogens frequently implicated in healthcare-associated infections [32,33]. Both species are known to form biofilms on abiotic surfaces, including medical devices, perpetuating healthcare-associated infections [34]. In this study, we explored the dynamics of biofilm formation in polymicrobial environments, focusing particularly on *S. aureus* and *P. aeruginosa*.

Polymicrobial biofilms are often more resilient to environmental changes and are also significantly more resistant to treatment options. The dynamics of polymicrobial biofilm interactions have previously been studied. Interestingly, contradictory results demonstrate the dynamic relationship between *S. aureus* and *P. aeruginosa*. In co-culture, *S. aureus* and *P. aeruginosa* have been shown to be antagonistic toward one another, while it has also been documented that *S. aureus* can persist through exposure to *P. aeruginosa*, even showcasing acquired vancomycin resistance [35,36].

Although these pathogens have been well-studied individually, their interactions in polymicrobial communities remain a complex and evolving area of research. Here, we showed that *P. aeruginosa* dominates when *S. aureus* and *P. aeruginosa* are cultivated together, suggesting an antagonistic relationship. Specifically, two strains of *P. aeruginosa* were capable of nearly eliminating the MRSA and MSSA biofilms.

We further demonstrated that not only is there an antagonistic relationship between *S. aureus* and *P. aeruginosa*, but secreted *P. aeruginosa* molecules can reduce *S. aureus* biofilms, like what has been previously reported [35,36]. Importantly, *P. aeruginosa* significantly reduces *S. aureus* biofilms by approximately 40%, suggesting that the exoproducts secreted are anti-staphylococcal. SEM analysis confirmed the disruption of biofilm structures in *S. aureus* treated with the CFCM from *P. aeruginosa*. Despite the significant impact observed on the *S. aureus* biofilm formation, there was a limited reduction in *S. aureus* planktonic growth in response to the *P. aeruginosa* CFCM. This indicates that the activity of the bioactive molecules produced by *P. aeruginosa* is stronger when *S. aureus* is present in a biofilm-favorable condition. Since bacterial cells are in a different metabolic state during planktonic growth than in biofilms, one hypothesis is that *P. aeruginosa* bioactive molecules depend on bacterial cell metabolism. Another possibility is that the activity is dependent on cell density, given that in a biofilm, cells are at a higher density compared with planktonic cells. Either way, this indicates that these bioactive molecules are specific to biofilm inhibition rather than causing general antimicrobial effects.

Furthermore, we demonstrated that in co-culture, *P. aeruginosa* excretes molecules, likely disrupting the integrity of the cell membrane of *S. aureus* during biofilm formation. This is not surprising as *P. aeruginosa* excretes LasA (~41 kDa), which is a known staphylolytic protein, cleaving pentaglycine bridges of peptidoglycan in the cell wall of *S. aureus* [37]. The excreted molecules are also potent in killing *S. aureus* cells, and are particularly effective against *S. aureus* biofilms, albeit with limited effect on planktonic bacteria. One limitation of this work is that the phenotypes were only analyzed after a 24-h incubation period. It is possible that a closer look at earlier time points would have provided additional information regarding the progression of the interaction between *P. aeruginosa* and *S. aureus* and the activity of the *P. aeruginosa* CFCM on different stages of the *S. aureus* biofilm. Nevertheless, our work provides a starting point for future studies to explore the dynamics of this interaction during different stages of biofilm formation.

Polymicrobial infections are increasingly recognized as complex clinical conditions where multiple microbial species interact within shared environments, often forming biofilms. These interactions significantly affect disease progression, antimicrobial susceptibility, and treatment outcomes. Interspecies interactions within these communities can alter the pharmacodynamics of antimicrobials. Herzberg and van Hasselt (2025) [38] demonstrated that interspecies interactions can modulate the pharmacodynamic response of pathogens to antibiotic treatments, usually having altered antimicrobial susceptibility profiles and growth rates. As the antimicrobial susceptibility is altered, it is important to understand the expression levels of key virulence factors involved in infections and biofilm formation, as this can dictate species dominance and susceptibility. To this end, we have shown that excreted molecules from *P. aeruginosa* alter the gene expression of key *S. aureus* virulence factors. We also showed that there was a decrease in expression for genes involved in attachment (*fib*) and the *agr* two-component system as well as an increase in the expression of *saeR*, which is responsible for the regulation of exoprotein production in *S. aureus* [28]. Unsurprisingly, when in co-culture, there was a marked increase in the exoprotein profile of *P. aeruginosa,* which is likely to be responsible for altering the growth rate and or the susceptibility profile of *S. aureus*, indicated by the lack of biofilm formation.

Through our fractionation, we discovered that no components contributing to the anti-staphylococcal activity were <10 kDa, suggesting that larger excreted compounds are responsible for this activity, which could include numerous pyocins, functioning as DNases, pore-formers, and RNases [18]. These excreted compounds allowed *P. aeruginosa* to dominate in the polymicrobial biofilm setting, which has important clinical applications, as recent work in cystic fibrosis patients demonstrated that co-infecting species can alter the antibiotic sensitivity of *P. aeruginosa* [39]. Although this work begins to address the question of how these species may interact within a clinical setting, namely in cystic fibrosis patients, further work is needed to better understand these interactions including identifying the active compounds responsible for the antagonism demonstrated here. In this work, we developed a framework whereby future studies will focus on the in-depth analysis of interaction to address the clinical efficacy of antimicrobials, and possibly help identify targets for therapeutic development, a key concern in polymicrobial biofilms [38,39].

## 4. Materials and Methods

### 4.1. Bacterial Strains

Three clinical isolates of *Pseudomonas aeruginosa* (CBAC 337, CBAC 338, and CBAC 394) were included in this study. CBAC 337 and CBAC 338 were isolated three days apart from each other from the ear secretion and tracheal aspirate of two different patients admitted at Antônio Pedro University Hospital (Niteroi, RJ, Brazil). CBAC 394 was isolated later from another patient from the same hospital. These three isolates, which shared very similar genotypic and phenotypic profiles, were selected to assess potential changes in their behavior during interactions with the *Staphylococcus aureus* strains used in this study. Two *S. aureus* strains were also included: the *agr*-defective RN4220 strain, provided by the Paulo de Góes Microbiology Institute at the Federal University of Rio de Janeiro (UFRJ), Brazil, and the methicillin-resistant USA300 (JE2) reference strain, obtained from the bacterial collection of the Laboratory of Molecular Epidemiology and Biotechnology at Fluminense Federal University (UFF).

All samples were cryopreserved in brain heart infusion (BHI, Kasvi, Parana, Brazil) broth with 15% glycerol and stored at −80 °C.

### 4.2. Polymicrobial Biofilm Assay

Bacterial strains were cultured in 10 mL tryptic soy broth (TSB—Kasvi, Parana, Brazil) for 4 h at 37 °C with shaking at 150 rpm. Then, 60 µL of inoculum was transferred into 12 mL of fresh TSB and cultivated for 14 h at 37 °C with shaking at 150 rpm. Cells were pelleted, washed twice, resuspended in sterile distilled water (dH_2_O), and the turbidity was adjusted according to McFarland standard 0.5 [~1.5 × 10^8^ colony forming units (CFU/mL)] at OD600 nm. The cultures were inoculated in 180 µL of TSB with 1% glucose in flat-bottomed 96-well plates (Nunc™, Thermo Fisher, Waltham, MA, USA) and mixed at a 1:1 inoculum ratio. The plates were incubated at 37 °C for 24 h (3 wells for each sample). After incubation, the biofilms were washed with phosphate-buffered saline (PBS), dried at 60 °C for 1 h, and stained with 0.1% crystal violet for 15 min. A total of 200 µL of 95% ethanol was added to measure the OD570 nm. For the enumeration of CFU/mL, biofilms were grown as described above with slight modifications. In brief, biofilms were cultivated on glass coverslips and grown overnight at 37 C for 24 h. The glass coverslips were placed in 10 mL of sterile saline solution, sonicated, and diluted 12 times in flat-bottomed 96 well-flat plates. Then, 10 µL of the dilutions were inoculated on TSA plates for general growth, on cetrimide agar plates for the isolation of *P. aeruginosa*, or on mannitol salt agar for *S. aureus* and incubated for 24 h at 37 °C. Colonies were then counted the next day to determine the CFU/mL.

### 4.3. Preparation of Cell-Free Conditioned Media (CFCM)

After overnight growth of *P. aeruginosa* CBAC 337 and CBAC 394 on tryptic soy agar (TSA—Sigma-Aldrich, St Louis, MO, USA), three colonies were inoculated into 50 mL of tryptic soy broth (K25—1224 TSB) and incubated for 24 h at 37 °C, shaking at 240 rpm. The cells were then centrifuged at 3100× *g* for 15 min, followed by filtration using a 0.22 µm pore size filter. The cell-free culture media (CFCM) was completely evaporated in a Speed Vac Concentrator (Labconco Centrivap, Kansas city, MO, USA). The sedimented residues were resuspended in sterile 0.85% NaCl to a concentration 20 times higher than the original [40]. The control extract underwent the same culture and treatment conditions, but without the addition of bacterial cells. To characterize the molecular weight of the compound responsible for the biological activity, the CFCM was partitioned using 3 kDa and 10 kDa molecular filters, evaporated in a Speed Vac, and resuspended in 0.85% saline solution as described above.

### 4.4. Determination of CFCM Activity on S. aureus Biofilm Formation

The biofilm assay was performed as described elsewhere [40,41]. Briefly, colonies grown overnight were transferred to sterile dH_2_O to achieve a turbidity relative to McFarland standard 0.5. From this suspension, 15 µL was inoculated into 120 µL of TSB supplemented with 1% glucose, along with 15 µL of CFCM at a 1:10 ratio in flat-bottomed 96-well plates and incubated for 24 h at 37 °C (3 wells for each sample). The supernatant was discarded after incubation, and the wells were washed twice with PBS. The plates were then incubated at 60 °C for one hour and stained with 0.1% crystal violet for 15 min at room temperature. The wells were washed twice with PBS, and 150 µL of 95% ethanol was added for 30 min. The OD570 nm was measured using a microplate reader (LOCCUS, LMR-96i).

### 4.5. Determination of CFCM Activity on S. aureus Planktonic Growth

Three colonies were inoculated into five mL of TSB for 24 h at 37 °C with shaking (240 rpm). Samples were diluted when the OD630 nm reached 0.05 in TSB containing either 10% CFCM or the control extract cultivated in 96-well plates (3 wells for each sample). Microplates were incubated at 37 °C with shaking (240 rpm), and the OD630 nm was recorded every 60 min for up to 24 h. All results confirmed were through CFU/mL enumeration.

### 4.6. Determination of CFCM Activity on S. aureus Biofilm by Scanning Electron Microscopy (SEM)

Colonies adjusted to a McFarland standard 0.5 were inoculated with 50 µL in 950 µL of TSB with 1% glucose and incubated in 24-well plates (Techno Plastic Products AG-TPP, Trasadingen, Switzerland) with glass coverslips, 13 mm, at 37 °C for 24 h (6 wells for each sample). For the treatment of *S. aureus* biofilms, 12% of CFCM was added to bacterial suspensions and mixed biofilms in a 1:1 ratio. The coverslips were washed 3 times with sterile saline solution before downstream processing steps. The coverslips were aseptically removed and treated with 25% glutaraldehyde for 1 h, then washed with a series of ethanol dilutions (10, 25, 50, 75, and 90%) for 20 min, and finally dehydrated with 99.5% ethanol for 1 h. The glass coverslips were incubated overnight at 37 °C and coated with gold in a low-pressure atmosphere. The biofilms were visualized using a scanning electron microscope (SEM, Carl Zeiss EVO, MA15, Oberkochen, Germany).

### 4.7. Determination of Total Colony-Forming Units Obtained from Biofilms

Biofilms cultivated on glass coverslips overnight were resuspended in 10 mL of sterile saline solution, sonicated, and diluted 12 times in flat-bottomed 96 well-flat plates. Then, 10 µL of the dilution was inoculated on TSA plates and incubated for 24 h at 37 °C. Colonies were counted the following day to determine the CFU/mL.

### 4.8. Gene Expression Analysis

*S. aureus* USA300 (JE2) cultures were grown overnight in 2 mL brain heart infusion (BHI) broth supplemented with 1% glucose with shaking at 220 rpm at 37 °C for 18 h. After 18 h, the bacterial suspension was diluted 1:100 in fresh BHI with 1% glucose and plated into a 24-well (Nunc™, Thermo Fisher, Waltham, MA, USA) plate with a final volume of 1 mL in each well. CFCM from CBAC 338 were added to each treatment well at a ratio of 1:10.

Plates were then incubated for 24 h at 37 °C. After 24 h, the supernatant was removed, and the attached bacteria were gently washed with 0.9% NaCl. The attached bacteria were scraped off the surface and resuspended in 0.9% NaCl and pelleted at 14,000× *g* for 2 min. To the pellet was added 100 µL of 1 mg/mL solution of lysostaphin in 1× Tris-Borate-EDTA (TBE) and this was then left to incubate for 10 min at room temperature. After lysis, the RNA was extracted using the Promega SV Total RNA isolation System following the recommended protocol.

RNA was quantified using a NanoDrop spectrophotometer. A total of 15.6 ng of RNA was used for reverse transcription. Reverse transcription and qPCR were conducted using the GoTaq^®^ 1-Step RT-qPCR Kit (Promega, Madison, WI, USA) following the manufacturer’s recommendations. Reverse transcription and amplification were carried out on an Applied Biosystems StepOne system (Thermo Fisher, Waltham, MA, USA). Standard cycling conditions were applied following the manufacturer’s recommendations. The relative gene expression was calculated using the 2^−ΔΔCt^ method using *gmk* as the internal control. Statistical significance was assessed using an unpaired *t*-test with Welch’s correction and two-way ANOVA using Šídák’s multiple comparisons test (significance *p* < 0.05) utilizing GraphPad Prism (v10.4.1).

### 4.9. Exoprotein Analysis

Bacterial pathogens were grown under the same conditions and sample setup as described in the Gene Expression Analysis section with one modification: bacteria were plated and grown in 2 mL of culture media in a 6-well plate. After 8 h, the supernatant was removed, and samples were normalized by OD_600_ measurement. Any remaining bacteria were then pelleted at 4000× *g* for 15 min and the supernatant collected. The collected supernatant was then run on a 4–20% TGX (Tris-glycine) SDS-PAGE gel (Bio-Rad, Hercules, CA, USA) and run at 100 V for ~120 min. The gel was washed 3 times with DI water for 5 min per wash and stained for 60 min using the Bio-Rad Coomassie safe stain (Bio-Rad, CA, USA). The gel was destained for 30 min in DI water. Gel images were analyzed using ImageJ by measuring the densitometry of the entire lane. The area under the curve was measured and plotted using GraphPad Prism (v10.4.1), and the statistical significance was assessed using an ANOVA (significance *p* < 0.05) with Dunnett’s multiple comparison test.

### 4.10. Laser Scanning Confocal Microscopy

Bacterial cultures were grown overnight in 2 mL of BHI broth with 1% glucose at 37 °C with shaking at 220 rpm. The bacterial cultures were then diluted 1:100 into fresh BHI broth with the addition of 1% glucose. For the polymicrobial biofilms, each species was diluted 1:100, ensuring that they were in a 1:1 ratio. The bacteria were then plated in a 24-well plate on 15mm sterile round glass coverslips (CellTreat, Ayer, MA, USA). The biofilms were then incubated for either four hours, eight hours, or 24 h. After incubation, the supernatant was removed, and the biofilms were washed with 20 mM HEPES buffer. After washing, the biofilms were stained with a 1 mg/mL solution of DiBAC4 (Ex: 493/Em: 516) (Invitrogen, Waltham, MA, USA) in ethanol and incubated for 15 min at room temperature. The coverslips were then washed once with nuclease-free water and stained for 30 min in 1 mL of water containing 5 µg/mL of Wheat Germ Agglutinin Alexa Fluor 647 (Ex: 650/Em: 665) (Invitrogen, MA, USA) and 5µM of SYTO84 (Ex: 567/Em: 582) (Invitrogen, MA, USA). Biofilm viability was carried out using the FilmTracer™ LIVE/DEAD^®^ Biofilm Viability Kit (Invitrogen, USA) according to the manufacturer’s recommendations. Coverslips were then mounted to clean glass slides (Fisher Scientific, MA, USA).

Images were acquired at Florida Atlantic University’s Advanced Cell Imaging Core using a Nikon A1R Confocal (Nikon, Tokyo, Japan) microscope mounted on a Ni-E microscope (Nikon, Tokyo, Japan). Images were acquired on a Plan Apo λ 60x Oil objective (Nikon, Tokyo, Japan) using 488, 561, 650 nm lasers with 0.1 µm z steps. Acquired images were processed using FIJI ImageJ by first splitting channels and then generating a PSF file using PSF generator using the Born and Wolf 3D Optical model. After the generation of a PSF model, image deconvolution was performed using DeconvolutionLab2 using the Richard–Lucy algorithm, using 10 iterations for each channel. Models were re-colored using lookup tables and selecting the appropriate color, and channels were merged. Brightness and contrast were adjusted by selecting the auto function applied to the entire image. Thresholding was applied to each Wheat Germ Agglutinin image, followed by defining cells based on thresholding. Cells identified through thresholding were then applied to the DiBAC4 images, and integrated density was used to calculate the fluorescence per cell [32]. Topological models of images were created using the 3D viewer plugin with volume selection.

### 4.11. Statistical Analysis

All experiments were performed at least three times using biological triplicates or biological duplicates where indicated (i.e., n = ). The mean of the triplicates for each sample was calculated using an analysis of variance (ANOVA) with a statistical significance level of 95% (*p* < 0.05) using Dunnett’s or Tukey’s post-test. A comparison between two conditions was performed using either a standard unpaired *t*-test or Welch’s *t*-test with a significance level of 95% (*p* < 0.05). Comparison between multiple groups in two conditions was conducted using two-way ANOVA using Šídák’s multiple comparisons test with a significance level of 95% (*p* < 0.05). All statistical analyses were performed using GraphPad Prism 10.4.1.

## 5. Conclusions

ESKAPE pathogens present a growing treatment challenge characterized by antimicrobial resistance, some of which are multidrug-resistant strains, further exacerbating and prolonging infections. *Staphylococcus aureus* and *Pseudomonas aeruginosa* are two well-known ESKAPE pathogens commonly found together in co-infections, leading to multi-microbial biofilm formation. These multi-microbial biofilms worsen clinical outcomes but remain understudied. We set out to address this gap by characterizing the early interactions between *S. aureus* and *P. aeruginosa* in a multi-microbial biofilm setting, focusing on the growth relationship and structure of multi-microbial biofilms. We started out investigating the dynamics of biofilm formation between *S. aureus* and *P. aeruginosa*, identifying an antagonistic relationship between the two species, although *P. aeruginosa*’s propensity to persist is observed through initial multi-microbial imaging (SEM). As we found that *P. aeruginosa* survives in a multi-microbial environment, further investigations demonstrated that cell-free culture media (CFCM) from *P. aeruginosa* could reduce biofilm formation and alter the structure of *S. aureus* biofilms. The activity of the CFCM was due to the larger molecular weight fraction (>10 kDa). Mechanistically, we have initially identified that a multi-microbial biofilm environment increases *P. aeruginosa* excreted proteins, which alter key biofilm-associated virulence factors in *S. aureus*. The factors *P. aeruginosa* excretes lead to membrane depolarization through treatment with CFCM and in a multi-microbial biofilm environment, reducing the overall cell viability. Overall, these results showcase an antagonistic relationship between two ESKAPE pathogens in a biofilm setting, highlighting the mechanism by which *P. aeruginosa* predominately survives in this relationship.

## Data Availability

The original contributions presented in this study are included in the article/Supplementary Materials. Further inquiries can be directed to the corresponding author(s).

## Supplementary Materials

The following supporting information can be downloaded at: https://www.mdpi.com/article/10.3390/antibiotics14050504/s1, Figure S1: Quantification of *S. aureus* and *P. aeruginosa* recovered from the polymicrobial biofilms; Figure S2: Quantification of the impact of the *Pseudomonas aeruginosa* cell-free conditioned media on the colony-forming units recovered from *Staphylococcus aureus* biofilms.

## Abbreviations

The following abbreviations are used in this manuscript:

ESKAPE	*Enterococcus faecium*, *Staphylococcus aureus*, *Klebsiella pneumoniae*, *Acinetobacter baumannii*, *Pseudomonas aeruginosa*, *Enterobacter* species
MDR	Multi-drug resistance
HQNO	2-n-heptyl-4-hydroxyquinoline-N-oxide
QS	Quorum sensing
SEM	Scanning electron microscopy
CFU	Colony forming units
MRSA	Methicillin-resistant *Staphylococcus aureus*
MSSA	Methicillin-susceptible *Staphylococcus aureus*
CFCM	Cell-free conditioned media
eDNA	Extracellular DNA
PI	Propidium iodide
