# Supplementary material for: In Vitro Analysis of Interactions Between Staphylococcus aureus and Pseudomonas aeruginosa During Biofilm Formation
Source: Antibiotics (Basel). 2025 May 14;14(5):504. doi: 10.3390/antibiotics14050504 (PMC12108489; doi:10.3390/antibiotics14050504)
Supplement: Supplementary file 1 [file antibiotics-14-00504-s001.zip › antibiotics-3590393-supplementary.pdf]

Figure S1.

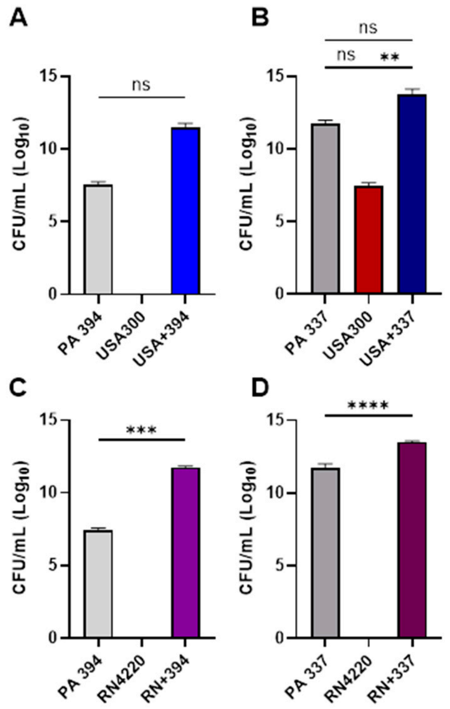

Quantification of *S. aureus* and *P. aeruginosa* recovered from polymicrobial biofilms. *S. aureus* USA300 cultivated with *P. aeruginosa* (A) CBAC 394 and (B) CBAC 337 and *S. aureus* RN4220 cultivated with *P. aeruginosa* (C) CBAC 337 and (D) CBAC 394. USA: *S. aureus* USA 300; 337: *P. aeruginosa* CBAC337; 394: *P. aeruginosa* CBAC 394; RN: *S. aureus* RN4220; ns, not significant. Polymicrobial biofilms tested include: USA300 + CBAC 394 (i.e., blue bar), USA300 + CBAC 337 (i.e., dark blue bar), RN4220 + CBAC 394 (i.e., purple bar), and RN4220 + CBAC 337 (i.e., plum bar). Statistical analysis was conducted using a Kruskal-Wallis one-way ANOVA with Dunn's multiple comparisons post hoc test with 95% ($p<0.05$) of significance or an Unpaired T-test. **: $p \leq 0.01$ (ANOVA); ****: $p \leq 0.0001$.

Figure S2

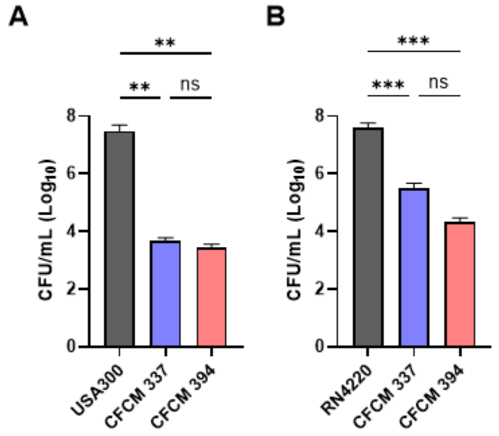

Quantification of the impact of *Pseudomonas aeruginosa* cell-free conditioned media on the colony-forming units recovered from *Staphylococcus aureus* biofilms. Colonies from *S. aureus* USA 300 biofilms without treatment and treated with *P. aeruginosa* CFCM 337 and CFCM 394 (A). Colonies recovered from *S. aureus* RN4220 biofilms without treatment and treated with *P. aeruginosa* CFCM 337 and CFCM 394 (B). CFCM: cell-free conditioned media; 337: *P. aeruginosa* CBAC337; 394: *P. aeruginosa* CBAC 394. Statistical analysis was conducted using an ordinary one-way ANOVA with Dunnett's post hoc test with 95% ($p<0.05$) of significance where: **: $p \leq 0.005$; ***: $p \leq 0.0005$.
